# Identification of core gene in obese type 2 diabetes patients using bioinformatics analysis

**DOI:** 10.1080/21623945.2021.1933297

**Published:** 2021-06-04

**Authors:** Zhiyong Dong, Xinyi Lei, Stacy A. Kujawa, NaciEmre Bolu, Hong Zhao, Cunchuan Wang

**Affiliations:** a Department of Bariatric Surgery, The First Affiliated Hospital of Jinan University, Guangzhou, China; b Feinberg School of Medicine, Robert H Lurie Comprehensive Cancer Center, Northwestern University, Chicago, IL, USA; c Department of Medicine, Istanbul University Istanbul Faculty of Medicine, Fatih, Turkey

**Keywords:** Obesity, type 2 diabetes, adipose tissue, core molecular markers, bioinformatics analysis

## Abstract

**Objectives** Adipocytes and adipocyte lipid metabolism are closely related with obesity and type 2 diabetes, but the molecular mechanism still needs further investigation. The aim of this study is to discover the adipocyte genes and pathways involved in obesity and type 2 diabetes using bioinformatics analysis.

**Methods** The GSE27951 gene expression profile was obtained. Software and online tools (STRING, Cytoscape, BioGPS, CTD, and FunRich) were used to identify core genes.21 human subcutaneous adipose samples, with 10 from type 2 diabetic patients and 11 from normal controls, were included in these analyses.

**Results** 184 differentially expressed genes (DEGs) including 42 up-regulated genes and 142 down-regulated genes were found to be enriched in metabolism, receptor activity, collagen type IV and glutamine biosynthesis I pathway by using the enrichment analysis. Seven hub genes were identified from the PPI network using various software (Cytoscape, STRING, BioGPS, and CTD). Four core genes (COL4A2, ACACB, GLUL, and CD36) were found to be highly expressed in subcutaneous adipose tissue of obese patients accompanying type 2 diabetes.

**Conclusion** COL4A2, ACACB, GLUL and CD36 might be the core molecular biomarkers of obesity in patients with or without type 2 diabetes.

## Introduction

Obesity is a serious chronic medical condition where patients have accumulated excess adipose tissue that could cause serious complications such as metabolic syndrome, type 2 diabetes mellitus (T2DM) and hypertension [1]. Studies have shown that obesity is increasing every year worldwide, with the number of adults with obesity increasing from 100 (81–122) million in 1975 to 671 (620–725) million in 2016 [2]. According to the statistics released by the International Diabetes Federation (IDF) in December 2015, there were about 450 million diabetic patients worldwide, who mainly suffered from T2DM; 75% were from developing countries [3,4]. Obesity and T2DM have become a burden globally as healthcare costs have increased with a growing population. Thus, determination of target genes promoting the pathogenesis of obesity and T2DMis vital for providing the future personalized treatment plans and offering timely preventative measures.

Obesity leads to accumulation of excess fatty tissues, such as the subcutaneous fat. Adipocytes are the major cell type in the fat tissue. Adipocytes and their metabolism are involved in the development of obesity and T2DM [5,6]. Research suggests that adiponectin is an insulin sensitizing hormone secreted by adipocytes that may promote the absorption of glucose, inhibit glycogen metabolism, stimulate the oxidation of adipocytes, and improve glycolipid metabolism disorders by binding to receptors of liver and skeletal muscle cells [7–9]. It has been reported that leptin, visfatin, and FGF21 play important roles in obesity and T2DM [10–12]. Moreover, Klimcakova E. et al. used mRNA and protein expression profiling techniques to confirm that adipose tissue macrophage-specific genes (e.g., TRAP, GP110, and M130) are markers of insulin resistance and the metabolic syndrome in human subcutaneous and visceral adipose tissue and thus play leading roles in adiposity [13]. However, it is still uncertain whether adipose tissue preferentially expresses core target genes of obesity and/or T2DM. The vast amount of the gene expression data deposited in public databases provides an opportunity to search for potential key genes of obesity and T2DM in adipose tissue. NCBI-Gene Expression Omnibus (GEO) is such a public database containing high-quantity gene expression data, such as gene chips, microarrays, and next generation sequence-based data [14]. With the gene data torrent from high throughput research in biology, the identification and analysis of differentially expressed genes (DEGs) is an important albeit massive undertaking. An integrated bioinformatics analysis, however, can be used to analyse these data for the discovery of human disease related DEGs.

In this study, we found gene datasets from human adipose tissue associated with obesity and/or T2DM in the GEO database and utilized the GEO2R web tool to define the groups and detect DEGs. We then used the Retrieval of Interacting Genes (STRING) online tool and Cytoscape software to analyse the DEGs and determine hub genes with a combined score of >0.4. Furthermore, the FunRich software was used to perform Gene Ontology (GO) and the pathway enrichment analysis. BioGPS, Comparative Toxicogenomic Database (CTD), and GEO2R were used to determine any correlation between diseases and expression of hub genes. Using all of these strategies, we found the potential gene markers associated with obese patients at risk for T2DM which may be useful for the personalized treatment of those epidemic diseases.

## METHODS

### Microarray data and patient sample selection

GEO (https://www.ncbi.nlm.nih.gov/geo/) is a public functional genomics data repository supporting array and next generation sequence-based data (including RNAseq, DNAseq, and ChIPseq) submissions. We searched the GEO DataSets using the keywords ‘diabetes’ AND ‘obesity’ AND ‘adipose’ to find related gene expression profiles. The gene expression profile of GSE27951 including 21 human subcutaneous adipose tissues regardless of T2DM status was obtained from the GEO database [14].

### Analysis and the identification of differentially expressed genes (DEGs)

GEO2R (https://www.ncbi.nlm.nih.gov/geo/geo2r/) is an interactive net software tool that allows operators to compare two or more different groups of samples in a GEO series to identify DEGs across experimental conditions via the GEO query and limma R packages (Bioconductor project). GEO2Rwas used to detect DEGs from different groups. We performed a t-test to identify DEGs with a P value <0.05. Log2-fold change (|logFC|) ≥2 cut-off was considered as statistical significance, with logFC≥2 considered as an up-regulated gene and LogFC ≤-2 considered down-regulated. The adjusted P values were used for multiple testing and genes with the smallest adjusted P-values were considered to be the most reliable. We also performed the distribution to see whether the selected samples were suitable for comparison by GEO2R where the median-centred values suggested that the data is normalized and cross-comparable. If not, samples were omitted.

### Integration of protein–protein interaction (PPI) network and modular analysis

STRINGv10.5 (https://string-db.org/) is a web tool used to calculate and evaluate the PPI information in order to expose the potential correlation among DEGs indifferent patient groups [15,16]. Following STRING analysis, we further utilized the Cytoscape software (V3.5.1, integrating biomolecular interaction networks with high-throughput expression data and other molecular states) to identify hub genes with k-core = 2, degree cut-off = 2and cut-off = 0.2. Confidence score of v ≥ 0.4 was considered significant. The related proteins in the central nodes were thought of as the core genes that have important biological regulatory functions [17].

### Comparison of the expression level of core genes on normal human adipose tissues by BioGPS

The BioGPS (http://biogps.org/#goto=welcome) is a free online extensible and customizable gene annotation portal tool, supplying a complete resource about gene and protein function. Based on HG_U133A/GNF1H and GNF1M Gene Atlas Data sets [18], we used BioGPS to identify the expression of newly identified core genes on subcutaneous and omental adipose tissues (E-GEOD-15,773 data, http://biogps.org/dataset/E-GEOD-15773/expression-data-from-human-adipose-tissue/). The barcode function of the R package ‘frma’ (http://www.bioconductor.org/packages/2.6/bioc/html/frma.html) was used to determine z-scores. A z-score of >5 implies that the gene was expressed in the tissue [19].

### Demonstration of the core genes associated with potential diseases

Comparative Toxicogenomic Database (CTD) (http://ctdbase.org/) is a public database that provides manually curated information about gene–disease relationships [20]. Based on the NCBI gene database (NCBI’s RefSeq project), we identified the diseases which were associated with each core gene by CTD.

### Functional enrichment and pathway analysis of hub genes

FunRich 3.0 (FR) is a software used to establish the functional enrichment of GO and pathways, gene expression, and interaction network analysis of genes and proteins [21]. FunRich software utilizes several databases (see: http://funrich.org/forum/faq). The data are integrated with functional and pathway data from GO. Pathway analysis via FR. P < 0.05 was considered as the cut-off criterion. We performed the GO analysis using FR with biological processes (BP), molecular functions (MF), cellular components (CC), and pathways among the hub genes [22]. The heat map used to show gene expression was generated by FunRich.

## Patient and public involvement

This bioinformatics’ research was conducted without patient and public involvement.

## Results

### Target samples and microarray information

The gene expression profile of GSE27951: ‘Adipo\genesis and obesity: subcutaneous adipose tissue’ was obtained from the GEO database. The microarray data fromGSE27951 (University of Stirling, United Kingdom was based on the GPL570 platform ([HG-U133_Plus_2] Affymetrix Human Genome U133 Plus 2.0 Array). Twenty-one homo sapiens subcutaneous adipose tissue obtained from 10 T2DM patients (three patients BMI >35 kg/m^2^, six patients BMI 25–34.9 kg/m^2^ and one patient BMI 18-24.9 kg/m^2^)) and 11 non-diabetes (NDM) (four patients BMI >35 kg/m^2^, four patients BMI 25–34.9 kg/m^2^, and three patients BMI 18–24.9 kg/m^2^) (Submission date: 14 2011; Last update date: 27 December 2017). Table 1 showed the characteristics of tissue information selected from GSE27951.

**Table 1.** The characteristics of patients information selected from GSE27951

Group	Gene Accession	Tissue Source	clinical status	BMI	Age	Fasting insulin	Fasting glucose	Hba1C
DM & BMI 35 kg/m^2^	GSM691125	SAT	DM	42.2	56	67	9.0	6.1
	GSM691129	SAT	DM	50.2	49	162	6.9	6.2
	GSM691144	SAT	DM	39.1	44	43	8.5	7.3
DM & BMI 25–34.9 kg/m^2^	GSM691142	SAT	DM	32.2	58	226	7.3	6.3
	GSM691138	SAT	DM	31.8	51	69	8.1	5.9
	GSM691141	SAT	DM	28.8	64	88	11.7	7.0
	GSM691137	SAT	DM	27.8	59	92	10.1	7.1
	GSM691133	SAT	DM	27.2	50	36	5.9	6.6
	GSM691148	SAT	DM	25.1	62	42	7.7	6.4
DM & BMI 18–24.9 kg/m^2^	GSM691136	SAT	DM	23.2	55	61	9.5	8.4
DM & BMI 35 kg/m^2^	GSM691122	SAT	NGT	38.1	22	117	5.2	5.5
	GSM691130	SAT	NGT	37.4	46	52	4.5	5.9
	GSM691131	SAT	NGT	36.7	45	59	5.0	5.7
	GSM691123	SAT	NGT	35.3	48	37	4.5	5.5
NDM & BMI 25–34.9 kg/m^2^	GSM691134	SAT	NGT	33.2	60	33	5.4	5.7
	GSM691124	SAT	NGT	32.9	39	62	5.0	5.7
	GSM691147	SAT	NGT	26.4	27	57	5.1	5.5
	GSM691150	SAT	NGT	26.2	57	19	4.8	5.5
NDM & BMI 18–24.9 kg/m^2^	GSM691143	SAT	NGT	23.6	37	35	5.5	5.5
	GSM691154	SAT	NGT	23.2	64	26	4.9	5.3
	GSM691153	SAT	NGT	23.1	42	28	5.1	5.4

Subcutaneous adipose tissue: SAT; GSE27951

### Identification of DEGs

**Figure 1.**
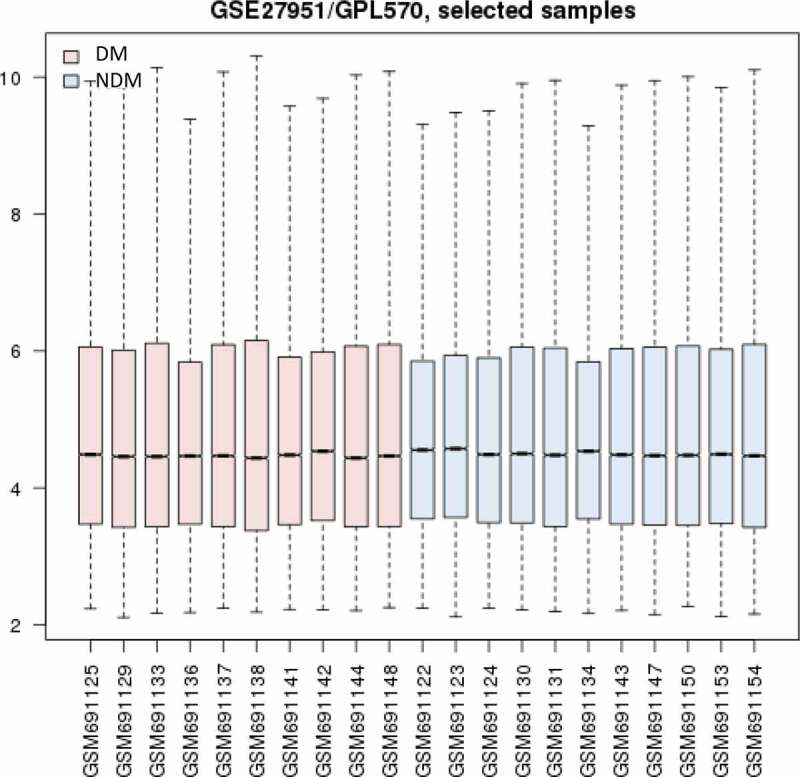
The median-centred values of included samples

The distribution of samples was viewed by GEO2R, the median-centred values are shown in Figure 1. It suggests that the data are normalized and cross-comparable, so the sample quality is reliable and suitable for comparison. These samples were cross-compared in BMI ≥35 kg/m^2^, BMI 25–34.9 kg/m^2^, and BMI 18–24.9 kg/m^2^ of NDM with T2DM patients (nine comparison groups in total), and the DEGs analysis was conducted in every group. The results showed that a total of 184 DEGs, including 42 up-regulated genes and 142 down-regulated genes, were found using GEO2R analysis. Based on BMI, T2DM and NDM patients were further divided into BMI ≥35 kg/m^2^, BMI 25–34.9 kg/m^2^ and BMI 18–24.9 kg/m^2^ (normal weight) group, respectively. We found 14 DEGs inT2DM with BMI ≥35 kg/m^2^ versus NDM with BMI ≥35 kg/m^2^ group, one DEG in T2DM with BMI 25–34.9 kg/m^2^ versus NDM with BMI 25–34.9 kg/m^2^ group, and 46 DEGs in T2DM-normal weight versus NDM-normal weight group. Within T2DM patients, we found two DEGs in BMI ≥35 kg/m^2^ versus BMI 24.9–35 kg/m^2^ group, 78 DEGs in BMI ≥35 kg/m^2^ versus normal weight group, and 16 DEGs in BMI 24.9–35 kg/m^2^ versus normal weight group. Within NDM patients, there were two DEGs in BMI≥35 kg/m^2^ versus BMI 24.9–35 kg/m^2^ group, 16 in DEGs in BMI ≥35 kg/m^2^ versus normal weight group and nine DEGs in BMI 24.9–35 kg/m^2^ versus normal weight group (selected by *P*. Value <0.05, ｜LogFC>2) ｜). Table 2 showed the detailed information of DEGs in nine comparison groups.

**Table 2.** The identification of DEGs in various groups

Groups	DEN	Differentially Expressed, Gene.symbol
BMI > 35 kg/m^2^:DM VS NDM	14	*Up-regulated*: MYH11, PLN, MYH11, SORBS2; *Down-regulated*: ANKDD1A, PCK1, FGF1, PEG10,MAL2, PHLDA2, TRDN, P2RY12 ERAP1, EGFL6
BMI 25–34.9 kg/m^2^:DM VS NDM	1	*Down-regulated*: CHI3L1
BMI 18–24.9 kg/m^2^:DM VS NDM	46	*Up-regulated*: EGFL6, LOC100509457///HLA-DQA2///HLA-DQA1, ABO, HLA-DRB4, ATP5E, LOC340107, HAMP, ELOVL7.*Down-regulated*: FAT3,MCOLN3,GLUL,TNN,PRKCD,ALPK3,AZGP1P1///AZGP1,ALDOC,MIR1908///FADS1,HILPDA,MYH2,RORB,ACACB,TMTC1,COL4A2,SIX1,CTBP1,MME,NRCAM,HLA-DQB1,OR51E1,PCNX1,STK26,COL6A6,PDE8B,PDE8B,PKD1L2,AZGP1,DEFB132,SPX
DM: BMI>35 kg/m^2^ VS 24.9–35 kg/m^2^	2	*Up-regulated*:PLN; *Down-regulated*:EGFL6
DM: BMI>35 kg/m^2^ VS 18–24.9 kg/m^2^	78	*Up-regulated*:ATP5E,SORBS2,ERAP1,PHLDB2,MS4A6A,FGF1,SULF1;*Down-regulated*:NMT2,REEP6,TFRC,ADAM12,MS4A7,COL11A1,IRF8,NQO1,IL1RN,MMP9,VGLL3,SIX1,PTPRC, #
DM: BMI 24.9–35 kg/m^2^ VS 18–24.9 kg/m^2^	16	*Up-regulated*:MYH2,TNNC2,NPR3,LOC101060835///LOC100996809///HLA-DRB6///HLA-DRB5///HLA-DRB4///HLA-DRB3///HLA-DRB1///HLA-DQB1,CTBP1,PKD1L2,TUFT1,MME;*Down-regulated*:ADGRG7,SMC3,HAMP,LOC340107,MALAT1,MALAT1,MALAT1,ATP5E
NDM: BMI>35 kg/m^2^ VS 24.9–35 kg/m^2^	2	*Up-regulated*:MALAT1;*Down-regulated*:HP
NDM: BMI>35 kg/m^2^ VS 18–24.9 kg/m^2^	16	*Up-regulated*:SPX,CA3,PCK1,RORB,AZGP1,GPAT3,MALAT1,PCNX1,DUSP4,TRDV3,GLUL;*Down-regulated*:IGHA2///IGHA1///IGH,MIR8071-2///MIR8071-1///IGHV4-31///IGHM///IGHG2///IGHG1,SFRP4,LOC100509457///HLA-DQA2///HLA-DQA1,EGFL6
NDM: BMI 24.9–35 kg/m^2^ VS 18–24.9 kg/m^2^	9	*Up-regulated*:HLA-DRB4;*Down-regulated*:RORB,YME1L1,LOC100509445///LOC728715///OVOS///OVOS2,DMRT2,SPX,PPP1R1B,MCOLN3,COL4A2

DEG: Differentially Expressed Genes No. DEGs were selected by PValue < 0.05; |logFC|> 2. DM:Type 2 Diabetes. NDM: None Diabetes

#:LOC100653057///CES1,LOC101060835///LOC100996809///HLA-DRB6///HLA-DRB5///HLA-DRB4///HLA-DRB3///HLA-DRB1///HLA-DQB1,ADAM12,MS4A6A,CTBP1,SLC1A4,BCAT1,CD63,PKD1L2,PHLDA2,CD163,SGK2,ITGB2,S100A8,FGF1,SLC41A2,ERAP1,RAB2A,HIST1H2AC,ABCC3,NRCAM,MIR675///H19,TUFT1,EGFL6,B4GALT6,COL11A1,NPR3,FCGR2A,GPLD1,LOC101930053///LOC101930048///VLDLR-AS1,MAL2,UBE2QL1,THBS1,TFPI2,MME,LOC100509457///HLA-DQA1,CHI3L1,DEFB132,P2RY12,HLA-DQB1

**Legends**: DM: type 2 diabetes. NDM: non-diabetes.

### Module screening from the PPI network

**Figure 2.**
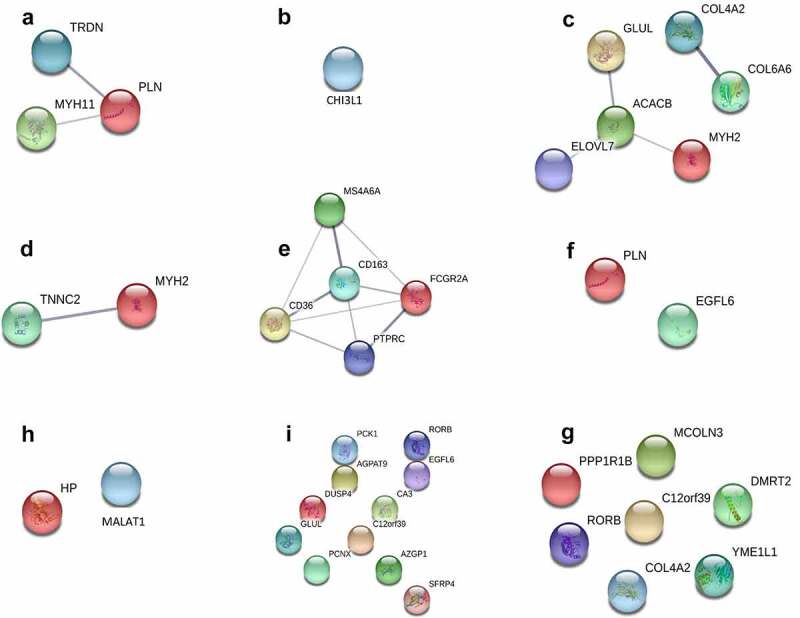
The interaction hub genes in different groups showed by STRING

We entered the DEGs of each comparison group and searched through the STRING database. The data was imported from the network as a simple tabular text using Cytoscape software to detect the hub genes. A combined score of>0.4 was considered statistically significant. Figure 2a-g showed the results of PPI analysis by STRING for interaction between the hub genes in four comparison groups: T2DM vs NDM in BMI ≥35 kg/m^2^ patients (Figure 2a); T2DM vs NDM in normal weight patients (Figure 2c); BMI ≥35 kg/m^2^ vs normal weight patients in T2DM patients (Figure 2d); and BMI ≥35 kg/m^2^ versus normal weight patients in NDM patients (Figure 2e). The results indicated that TRDN, PLN, and MYH11 were potential hub genes for the DM versus NDM in BMI ≥35 kg/m^2^ patients group (Figure 2a), GLUL, ELOVL7, MYH2, ACACB, COL4A2 and COL6A6 were potential hub genes for DM versus NDM in normal weight patients (Figure 2c), TNNC2 and MYH2 were potential hub genes for BMI 24.9–35 kg/m^2^ versus normal weight group in DM patients (Figure 2d), and MS4A6A, CD36, PTPRC, FCGR2A and CD163 were potential hub genes for BMI ≥35 kg/m^2^ versus normal weight in T2DM patients (Figure 2e). We did not find potential hub genes when we compared T2DM versus NDM in BMI 24.9–35 kg/m^2^ patients (Figure 2b, F, H, I and G). Because MYH2 was the same gene in the two groups, so taken together, TRDN, PLN, MYH11, COL4A2, COL6A6, ACACB, GLUL, ELOVL7, CD36, FCGR2A, PTPRC, CD163, MS4A6A, TNNC2 and MYH2, 15 genes were considered as hub genes.

**Legends**: (A) BMI >35 kg/m^2^: DM versus NDM group. (B) BMI 25–34.9 kg/m^2:^ normal weight: DM versus NDM group. (C) BMI 18–24.9 kg/m^2^: DM versus NDM group. (D) DM: BMI ≥35 kg/m^2^ versus 24.9–35 kg/m^2^ group. (E) DM: BMI > 35 kg/m^2^ versus normal weight group. (F) DM: BMI 24.9–35 kg/m^2^ versus normal weight. (H) NDM: BMI > 35 kg/m^2^ versus 24.9–35 kg/m^2^ group. (I) NDM: BMI > 35 kg/m^2^ versus normal weight group. (G) NDM: BMI 24.9–35 kg/m2 versus normal weight group.

### The potential hub gene expression levels in normal tissues

**Figure 3.**
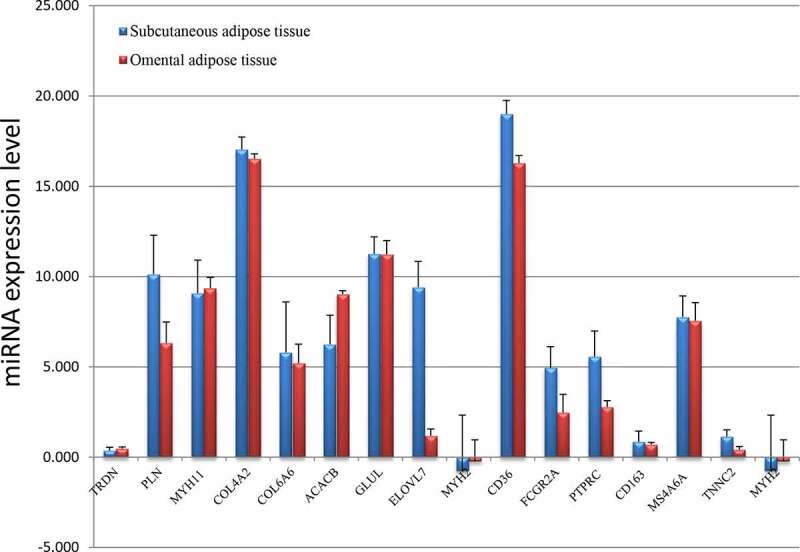
The expression level of hub genes on normal human adipose tissues, using by BioGPS

**Figure 4.**
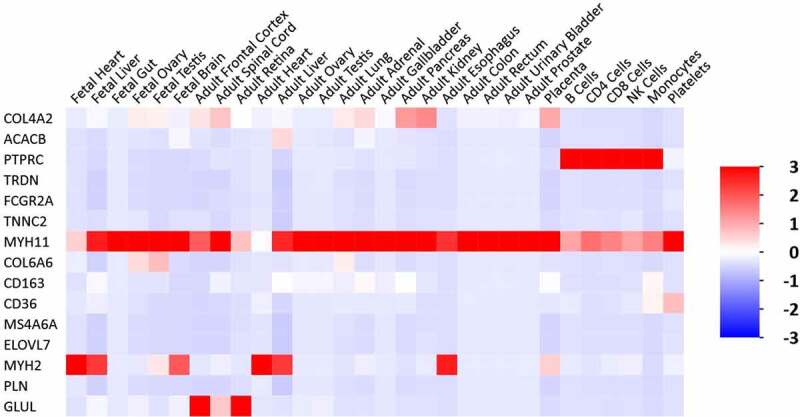
Heatmap showing log2 transformed ratios of hub genes in normal human organ or cell lines by FunRich

We used BioGPS to identify the hub gene expression in normal tissues (Dataset: Barcode in 262 normal tissue samples; Probe set: 242194_×_at). This data set demonstrated a survey across various normal human tissues (platform: U133plus2 Affymetrix microarray). The median, 3X median, 10X median, and 30X are defaults of the BioGPS presentation sketched by the lines and are not central to this analysis. The results showed that COL4A2, GLUL and CD36 were highly expressed both in insulin resistant omental adipose tissue and subcutaneous adipose tissue, and PLN, ACACB, and ELOVL7 were highly expressed in insulin resistant subcutaneous adipose tissue (Figure 3). Figure 4 showed the expression level of hub genes in normal human organs by FunRich. Table 3 illustrates the expression level of hub genes in normal human adipose tissues.

**Table 3.** The hub genes miRNA expressed in normal adipose tissue using BioGPS and related disease by CTD

Groups	Hub genes	Adipose tissue	Adipose tissue omental	Related Disease	Inference Score
BMI > 35 kg/m^2^:DM VS NDM	TRDN	0.37 ± 0.19	0.48 ± 0.09	Weight Loss	37.25
	PLN	10.13 ± 2.16	6.33 ± 1.16	Glucose Intolerance	37.13
	MYH11	9.08 ± 1.83	9.36 ± 0.60	Weight Loss	100.69
BMI 18–24.9 kg/m2:DM VS NDM	COL4A2	17.04 ± 0.69	16.53 ± 0.27	Weight Loss	92.25
	COL6A6	5.80 ± 2.80	5.21 ± 1.05	Diabetes Mellitus	16.00
	ACACB	6.25 ± 1.61	9.03 ± 0.19	Insulin Resistance	85.45
	GLUL	11.26 ± 0.94	11.23 ± 0.77	Weight Loss	175.47
	ELOVL7	9.41 ± 1.43	1.19 ± 0.37	Weight Loss	77.78
	MYH2	−0.76 ± 3.09	−0.23 ± 1.19	Insulin Resistance	40.55
DM: BMI>35 kg/m2 VS 18–24.9 kg/m2	CD36	19.01 ± 0.75	16.29 ± 0.41	Weight Loss	239.26
	FCGR2A	4.96 ± 1.16	2.48 ± 1.00	Weight Loss	34.41
	PTPRC	5.57 ± 1.42	2.78 ± 0.35	Weight Loss	84.92
	CD163	0.86 ± 0.59	0.7 ± 0.12	Weight Loss	98.07
	MS4A6A	7.76 ± 1.17	7.56 ± 1.00	Glucose Intolerance	33.85
DM: BMI 24.9–35 kg/m2 VS 18–24.9 kg/m2	TNNC2	1.14 ± 0.37	0.42 ± 0.17	Weight Loss	70.86
	MYH2	−0.76 ± 3.09	−0.23 ± 1.19	Insulin Resistance	40.55

CTD: comparative toxicogenomics database; BMI: Body mass index; DM: type 2 diabetes; NDM: non-diabetes. mean± SD: miRNA expression

Legends: Blue sharp is subcutaneous adipose tissue. Read sharp is omental adipose tissue.

Legends: the rectangle of right side is fold change value (log), red means positive correlation; blue means negative correlation.

### Demonstration of the potential hub genes associated with potential diseases

To identify the diseases that are most associated with hub genes, we input the potential hub genes to CTD for defining the greater the inference score of the individual gene, which means the more relevance with disease. The CD36, GLUL, MYH11, CD163, and COL4A2 genes were closely associated with weight loss, while the ACACB gene was closely related to insulin resistance (Table 3). The results also showed that PLN was less relevant to glucose intolerance. The heat-map generated by EnRich showed the expression level of the potential hub genes in normal human organs, with MYH11 having the highest expression in every normal organ. However, MYH11 is a non-specific gene and thus was not considered as a hub gene in this study (Figure 4). Taken together, CD36, GLUL, COL4A2, and ACACB were considered as core genes in human adipose tissue with obesity or T2DM.

### The outcome of function and pathway enrichment analysis

In order to identify the function and pathway of the potential hub genes, GO function and pathway enrichment analyses were applied by using FunRich. CD36, GLUL, COL4A2, and ACACB were put into FR software, the gene enrichment analyses showed that hub genes enriched in biological processes (BP), including metabolism, cell growth and/or maintenance, and cell communication. Molecular function (MF) includes receptor activity, ion channel activity, and transaminase activity. Cell component (CC) includes collagen type IV, Golgi apparatus, and platelet alpha granule membrane). The significantly enriched biological pathways include neurotransmitter uptake and metabolism in glial cells, astrocytic glutamate-glutamine uptake and metabolism, and glutamine biosynthesis I (Table 4).

**Table 4.** GO and pathway enrich analysis of hub gene associated with obesity type 2 diabetes

Enrichment terms	No. of genes in the dataset	No. of genes in the background dataset	Percentage of genes	Fold enrichment	P-value (Hypergeometric test)	Q-value (Storey-Tibshirani method)	Genes
**Biological process**							
Metabolism	3	1683	42.85714	4.906155	0.017897	1	CD36;GLUL;ACACB;
Cell growth and/or maintenance	2	1125	28.57143	4.901186	0.059	1	COL4A2;
Cell communication	2	3713	28.57143	1.485017	0.40418	1	CD163;
**Molecular function**							
Transaminase activity	1	23	14.28571	120.411	0.008344	1	GLUL;
Ion channel activity	1	66	14.28571	41.9733	0.023783	1	PLN;
Receptor activity	2	361	28.57143	15.27349	0.006934	1	CD36; CD163;
**Cellular component**							
Golgi aparatus	3	897	42.85714	9.205146	0.003074	1	CD36;GLUL;ACACB;
Platelet alpha granule membrane	1	11	14.28571	251.6492	0.003998	1	CD36;
Collagen type IV	1	5	14.28571	553.0255	0.001819	1	COL4A2;
**Biological pathway**							
Neurotransmitter uptake and Metabolism In Glial Cells	1	2	14.28571	1378.437	0.000728	1	GLUL;
Astrocytic Glutamate-Glutamine Uptake And Metabolism	1	2	14.28571	1378.437	0.000728	1	GLUL;
glutamine biosynthesis I	1	1	14.28571	2743.225	0.000364	1	GLUL;

## Discussion

It is estimated that 493 million people will be either obese or overweight by 2030, and 629 million people will be diabetic by 2045 worldwide [23]. It will consume enormous resources and cause immense challenges to maintain well-being in a population with the majority being obese or overweight [24]. Dietary adjustment, medication, and/or surgical intervention can help to obtain certain weight loss achievements, but personalized treatment by identifying key genes or pathways relating obesity and diabetes may provide major improvements in the treatment of obesity and T2DM. Obesity and T2DM are closely related to similar genetic, epigenetic, adipose, metabolism, and endocrine aberrations [25]. Understanding the molecular mechanism behind obesity and T2DM is critically important for early diagnosis and precise treatment. Therefore, it is vital to discover these sensitive and specific gene biomarkers of obesity or T2DM.

In this analysis, 21 subcutaneous adipose tissues were extracted from the GEO database of GSE27951. Then, bioinformatics analysis was performed to identify candidate genes from biological database. It is useful for understanding the differences between expressed genes, potential hub genes, pathways, genetics, or unique adaptations amongst samples. Forty-two up-regulated and 142 down-regulated DEGs were selected. Then, TRDN, PLN, GEFL6, MYH11, COL4A2, COL6A6, ACACB, GLUL, ELOVL7, MYH2, CD36, FCGR2A, PTPRC, CD163, CD36, MS4A6A, TNNC2, and MYH2 were considered as hub genes by bioinformatics analysis. It was shown that no hub genes were found in NDM groups (BMI ≥35 kg/m^2^ versus BMI 25–34.9 kg/m2, BMI ≥35 kg/m^2^ versus normal weight, and BMI 25–34.9 kg/m^2^ versus normal weight), suggesting that there were no differences among those NDM samples with different BMIs [26]. The lack of DEGs in the BMI ≥35 kg/m2 group (DM versus NDM) indicated that if there are more DEGs between DM and the NDM, the greater differential BMI values will be. Obesity is a risk for diabetes as the incidence of diabetics with obesity is more than those with diabetes who are overweight and those with diabetes who are normal weight [23]. Here BioGPS, CTD, FunRich, GO and pathway analysis were used to identify the potential hub genes. As a result, CD36, COL4A2, GLUL, and ACACB were selected and considered as hub genes that may provide novel targets for further studying obese diabetic patients.

The GO and pathway analysis showed that CD36 enriched biological function (FP, MF, CC) in metabolism, receptor activity, and platelet alpha granule membrane where the enriched pathway exists due to platelet adhesion to exposed collagen. CD36 is member 3 of the scavenger receptor class B family of cell surface proteins and is located on the long arm of chromosome 7 at band 11.2 in humans. It may play a key role in the development of glucose intolerance and diabetes [27,28]. Tahar et al. reported that, in a low fat and high starch diet, CD36 deficiency enhanced insulin responsiveness in CD36-null mice [29]. Pravenec et al. reported that transgenic expression of CD36 was closely associated with reduced serum fatty acids as well as improvement of insulin resistance and glucose intolerance in the spontaneously hypertensive transgenic rat models [30]. Drover et al. indicated that CD36 deficiency is a possible risk factor for diet-induced T2DM in both the postprandial and fasting states in humans [31]. Our results also showed that CD36 was more highly expressed in T2DM with normal weight than T2DM with obesity. In addition, CD36 was closely correlated with weight loss (analysed by GEO2R), suggesting that a decreased CD36 levels may lead to an increase in body weight.

The GO and pathway analysis showed that GLUL enriched the biological functions (FP, MF, CC) of metabolism and transaminase activity in the Golgi apparatus, and its closest related pathway is glutamine biosynthesis I. GLUL (ligase) is an enzyme-coding gene that belongs to the glutamine synthetase family [32,33]. GLUL plays a crucial role in the metabolism of the nitrogen form of glutamine in an ATP-dependent reaction [32]. Our study also showed that GLUL had a higher expression in non-diabetic normal weight patients than in diabetic normal weight patients; GLUL was also closely correlated with weight loss. Petrus et al. also reported that GLUL expression was lower in the obese group with human white adipose tissue than non-obese group [34]. This observation suggests that increasing GLUL levels may help to reduce diabetes. Prudente et al. reported that the rs10911021 polymorphism of the GLUL genies an independent modulator of mortality in T2DM patients [35]. The Look AHEAD Research Group performed genetic analyses in 3,845 overweight/obese participants with T2DM over a median of 9.6 years. The results showed that the risk (C) allele for GLUL rs10911021 was significantly associated with morbidity and mortality from cardiovascular disease among T2DM patients [36].

COL4A2 was found to enrich the biological functions (FP, MF, CC) of cell growth and/or maintenance as well as the expression of extracellular matrix structural constituents and collagen type IV (COL4). However, no pathways were enriched. COL4 Alpha 2 (COL4A2) chain is the encoded basement membranes by the COL4A2 gene that exist in humans [37]. Du et al. suggested that the reduction of miR-29a caused by high glucose may increase the risk of excess COL4A2 in proximal tubule cells [38]. This study showed that COL4A2 is highly expressed in adipose tissue, which is related to weight loss. We plan to examine the relationship between COL4A2 expression and the pathogenesis and progression of T2DM and obesity in future studies.

ACACB (Acetyl-CoA carboxylase β) was found to enrich the biological functions (FP, MF, CC) of metabolism, ligase activity, and the Golgi apparatus and its closest related pathway is the import of palmitoyl-CoA into the mitochondrial matrix. ACACB is an enzyme that associated with diseases including Acetyl-Coa Carboxylase-Beta Deficiency and Biotin deficiency and it is related with the glucagon signalling pathway. ACACB is thought to control fatty acid oxidation through malonyl-CoA to inhibit carnitine palmitoyl transferase I, which is the rate-limiting step in fatty acid uptake and oxidation by mitochondria [39]. Our study also showed that ACACB had higher expression in non-diabetic normal weight patients compared to diabetic patients with normal weight. It also showed that ACACB was closely correlated to insulin resistance. This denotes that increasing ACACB levels may help to reduce diabetes. MA et al. showed that ACACB plays a role in obesity-altered lipid metabolism in susceptibility to T2DM [40]. An et al. analysed 12 case-control studies containing 3273 cases and 3242 controls, which indicated that there are significant associations between the ACACB gene, rs2268388 polymorphism, and diabetic nephropathy among Caucasian patients with diabetes. Some case reports also suggested that ACACB expression may be related to obesity [41]. For example, the Corbett et al. study used ACACB as a target in the design of isozyme-non-selective acetyl-CoA carboxylase inhibitors in obese mice [42]. After analysing the array dataset, GSE29718 [43], it is concluded that immune system pathways may have a significant role in child obesity. In contrast, Wei et al. reported that ACACB polymorphisms were associated with blood pressure in an analysis of Han Chinese T2DM populations from 1975 [44]. In a twin study, ACACB polymorphisms in biotin-dependent carboxylases were found to be down-regulated in adipose tissue and adipocytes of the obese twin in comparison with the non-obese twin. Taken together, CD36, COL4A2, GLUL, and ACACB were considered as core genes closely related to obesity and T2DM.

## Limitation

First, the sample size of each group in this study was limited, and regarding the inevitably bias from the heterogeneity of clinical tissues, more other databases containing tissues from obesity or T2DM should be included further. Second, the gender and age should be considered as well. Third, the adipose tissues from different parts of the body, including subcutaneous adipose, the omentum, jejunal mesentery, and ileal mesentery, as well as the adipose near to the lesser curvature and greater curvature of stomach of obese or overweight patients, should be utilized to confirm those core genes.

## Conclusions

CD36, COL4A2, GLUL, and ACACB that are significantly enriched in metabolism and glutamine biosynthesis I, astrocytic glutamate-glutamine uptake, and neurotransmitter uptake and metabolism in glial cells. They are also related to weight loss and insulin resistance. These genes might be the core molecular biomarkers for obesity or T2DM. Furthermore, these results provided strong evidence for forthcoming therapeutic research involving precise gene targets in T2DM and NDM obese patients. We anticipate identifying the genes closely related to obesity and T2DM and further investigate their relationship with the CD36, COL4A2, GLUL, and ACACB as they may be target genes for future therapies.

## Data Availability

The RNA microarray data that support the findings of this study are from previously reported and public available studies and the NCBI Gene Expression Omnibus (GEO) datasets, which have been cited.

## Article highlights

- With the gene data torrent caused by high throughput research in biology, the identification of differentially expressed genes and utilization of the information garnered from genes is vital information albeit a massive undertaking
- In order to make sense of these data, researchers use an integrated bioinformatics analysis, which may also provide valuable evidence for further research
- This study confirmed that COL4A2, ACACB, GLUL and CD36 may be the core molecular biomarkers of obesity in the subcutaneous adipose tissue of patients with diabetes

## Statement of Human and Animals Rights

There is no animal and human experiment involved in the study.

## Open access

This is an open access article distributed in accordance with the Creative Commons Attribution Non Commercial (CC BY-NC 4.0) license, which permits others to distribute, remix, adapt, build upon this work non-commercially, and license their derivative works on different terms, provided the original work is properly cited, appropriate credit is given, any changes made indicated, and the use is non-commercial. See: http://creativecommons. org/licenses/by- nc/4. 0/.
